# Correlation of Patterns of Bone Marrow Infiltration and Biochemical factors in Non-Hodgkin Lymphoma

**DOI:** 10.12669/pjms.332.11335

**Published:** 2017

**Authors:** Naghmana Mazher, Nisar Ahmad, Zafar Iqbal

**Affiliations:** 1 Dr. Naghmana Mazher, Post Graduate Resident FCPS Part II. Department of Pathology, The Children’s Hospital and Institute of Child Health, Lahore, Pakistan; 2 Dr. Nisar Ahmad, FCPS. Head of Hematology and Transfusion Medicine, Department of Pathology, The Children’s Hospital and Institute of Child Health, Lahore, Pakistan; 3 Dr. Zafar Iqbal, M.Phil. Additional Director, Blood Transfusion Centre, Punjab, Lahore, Pakistan

**Keywords:** Non- Hodgkin Lymphoma, Beta 2 microglobulin, Lactate Dehydrogenase

## Abstract

**Objectives:**

To investigate the patterns of bone marrow involvement in in Non-Hodgkin’s Lymphoma (NHL) patients and to correlate the patterns with β_2_ microglobulin (β2m) and lactate dehydrogenase 2 (LD2) levels in these patients.

**Methods:**

It was a cross sectional study which was conducted in two years at Post Graduate Medical Institute, Lahore and at Centre of Excellence in Molecular Biology (CEMB), Lahore. The study was conducted on 50 subjects irrespective of age and sex divided into two groups i.e. Group-A comprising 20 normal healthy controls while Group-B consisted of 30 patients of NHL with bone marrow infiltration. Bilateral bone marrow trephine biopsy was done to assess the patterns of bone marrow infiltration. Serum β2m and LD2 isoenzyme levels were determined in already diagnosed 30 patients of NHL. The values were compared with 20 healthy age and sex matched controls. Correlation coefficients were determined using Pearson’s Correlation Coefficient. The estimations were made prior to the institution of chemotherapy.

**Results:**

β2m and LD2 levels were significantly (p-Value < 0.05) raised in NHL patients with disease advancement and were compared with controls. These serological markers showed negative correlation (-0.235 for β2m and -0.133 for LD2) with the spread of disease and patterns of involvement in NHL patients.

**Conclusion:**

By observing the patterns of bone marrow involvement in NHL patients possible guidelines about prognosis and treatment protocols can be obtained as the serological markers levels correlate well with the spread of disease and patterns of involvement in NHL patients.

## INTRODUCTION

Lymphomas represent clonal malignancies of lymphoreticular system in which the majority of the cells are frozen at a single stage of normal differentiation.^1^ Two broad types of lymphomas are named as HD and NHL.^2^

Cancer is a group of over 100 diseases, all of which start with the growth of abnormal cells. Instead of dying in the normal cell life cycle, cancerous cells continue to divide into new abnormal cells, and grow out of control.^3^ The malignant behavior such as how aggressive or slow growing it might be is determined by cell type and pattern, a kind of mutation and sometimes by host environment. Cell types are primarily determined by histology. The size, nuclear and cellular features of lymphoma cells allow the identification of lymphoma by its appearance and its markers. The pattern by which a lymphoma infiltrates and replaces a previously normal lymph node is predictive of its biological behavior. The architectural pattern of proliferative process that of either a diffuse or a follicular appearance is of prognostic value. In general follicular pattern has a better prognosis than diffuse pattern.^3^,^4^ Incidence of marrow disease varies with histological subtype of NHL. Bone marrow trephine biopsy is important in identifying marrow involvement for treatment purpose.^5^

Histological patterns can be assessed on the sections of trephine biopsies. Any of the three patterns i.e. interstitial, focal and diffuse can be seen either alone or in combination.^6^,^7^ Patients at high risk for failure with conventional therapy may benefit from investigational approaches. The biological markers of NHL are distinguished in three categories: serological, immunophenotypic and molecular markers. Among the most important serological markers β_2_m reflects the tumor load and LD indicates invasive potentials of lymphoma.^8^

β_2_m is a low molecular weight polypeptide, non-covalently linked to the heavy chain of class 1- histocompatibility antigens which are shed with cell turnover. It is plentiful on the surface of lymphocytes. Increased production or destruction of the cells cause β_2_m levels in the blood to increase.^9^ LD has molecular weight of 135,000 Daltons. It is a zinc containing enzyme. LD catalyzes the reversible oxidation of lactate to pyruvate. It is expressed at higher levels when lymphocytes are dividing or when cells are distressed or damaged. Elevating LD is an indication of disease progression. Sharp increase can indicate transformation. LD has five isoenzymes which differ slightly in structure. LD2 is concentrated in lymphocytes.^10^,^11^

This study was conducted to investigate the patterns of bone marrow involvement in NHL patients and to correlate these patterns with β2m and LD2 levels in these patients.

## METHODS

It was a cross sectional study conducted on 50 subjects irrespective of age and sex divided into Group A comprising 20 normal healthy controls and group B consisting of 30 patients of NHL with bone marrow infiltration.

The following formula was used for estimating the sample size.


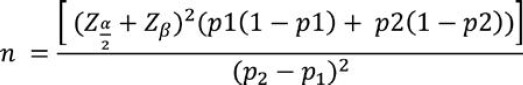


With following Assumptions:

P1 = Expected Prevalence of infiltration of NHL with bone marrow=12.4%

P2 = Expected Prevalence of infiltration of NHL without bone marrow=30%

|p2-p1| (i.e., the minimum expected difference)

A significance criterion of 5% (0.05) and a power of 80% (0.80) were chosen.

Level of Significance= α= 5%

Z_α/2_= for 95% confidence level =1.96

Power of the Test= 1-β=80%

Z_α/2_= 80% power of study = 0.84

Hence, the minimum sample size is calculated as below.

n =Total Sample Size: 50 patients

The total no. of subjects (Total Sample Size) was distributed into two groups for inferential statistics.

Number of control patients = 20

Number of NHL patients = 30

Total number of patients = 50

The sample size for two groups was designed as under:

**Controls (Group A)**: In this group 20 Normal healthy subjects (controls) were enrolled.

**Group B**: In this group 30 patients of NHL with bone marrow were enrolled.

Non-probability, purposive sampling technique was used.

The cases were selected from Lahore General Hospital, Lahore, Institute of Nuclear Medicine and Oncology (INMOL), Lahore, Services Hospital, Lahore and Mayo Hospital, Lahore.

### Inclusion and Exclusion criteria

Newly diagnosed cases of NHL by lymph node biopsy prior to the institution of chemotherapy of both sexes and all age groups were selected for the present study. The patients with the history of myocardial infarction, renal failure, hepatic dysfunction, skeletal muscle disease, hemolytic anemia, malignancy of any other system, cerebrovascular accident, Infectious mononucleosis and intestinal infarction were excluded.

Bilateral bone marrow trephine biopsy was done bilaterally from right and left posterior superior iliac spine to assess the patterns of bone marrow infiltration. Among serological markers β2m was estimated by Enzyme Linked Immunosorbent Assay (ELISA). LD2 was measured by Agarose Gel Electrophoresis at Centre of Excellence in Molecular Biology (CEMB), Lahore. The results were analyzed by using Student’s ‘t’ test with 95% confidence level. P-value < 0.05 was taken statistically significant. The statistical methods were used to calculate arithmetic mean, standard deviation, probability ‘P’ value, correlation and using Pearson’s Correlation Coefficient. These calculations were carried out using SPSS V16.0.

### Ethical consideration

Informed verbal consent was taken prior to the interview. The respondents were informed about the purpose of the study. The confidentiality of the information was ensured and maintained.

## RESULTS

In NHL patients diffuse pattern of bone marrow infiltration was the commonest being observed in 14 (46%) cases followed by interstitial infiltration in 10(33%) cases. In the focal variety, paratrabecular pattern was present in 4(13%) patients while random type was observed in 2(6%) cases (Table-I). As bone marrow biopsy was performed bilaterally, the yield of positive cases of bone marrow infiltration was increased. The diffuse pattern was seen in nine cases unilaterally and in 5 cases bilaterally. As regards interstitial pattern it was observed unilaterally in seven cases and bilaterally in 3 cases while paratrabecular pattern was seen in three cases unilaterally and in one case bilaterally. Random pattern was observed in two cases unilaterally.

**Table-I:** Mean values of β2m and LD2 Levels in Patterns of Bone Marrow Infiltration.

*Bone Marrow Patterns*	*No. of Patients (n=30)*	*Mean β2m (µg/ml)*	*Mean LD2 (%)*
Diffuse	14(46%)	4.2 ± 0.84	55.07 ± 2.97
Interstitial	10(33%)	3.87 ± 0.49	52.1 ± 4.99
Paratrabecular	4(13%)	3.47 ± 0.28	48.5 ± 5.06
Random	2(6%)	3.05 ± 0.07	45.5 ± 6.36

In patients with diffuse pattern mean β2m level was 4.2 ± 0.84, in interstitial was 3.87 ± 0.49 while paratrabecular and random had 3.47 ± 0.28 and 3.05 ± 0.07 µg/ml respectively and the LD2 values in diffuse, interstitial, paratrabecular and random pattern were 55.07 ± 2.97, 52.1 ± 4.99, 48.5 ± 5.06 and 45.5 ± 6.36% respectively (Table-I).

Comparing β2m and LD2 levels among controls and Bone Marrow infiltration NHL patients was statistically highly significant (P-Value < 0.05) with negative correlation values for β2m and LD2 was -0.235 and -0.133 respectively (Table-II).

**Table-II:** β2m and LD2 levels in controls and NHL patients with bone marrow infiltration.

*Parameters*	*Controls(n = 20)*	*Infiltration(n = 30)*	*P value*	*Pearson Correlation Coefficient (r)*
β2m(µg/ml)	1.52 ± 0.43	3.93 ± 0.71	<0.05*	-0.235
LD2 (%)	28.85 ± 4.107	52.53 ± 4.967	<0.05*	- 0.133

* Very highly significant (Using t-test)

**Fig.1:**
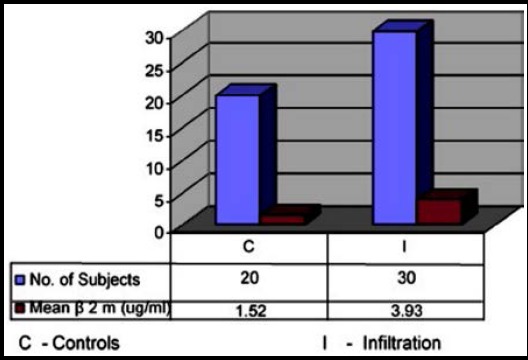
Comparison of mean values of β2m in controls and NHL patients with marrow infiltration.

**Fig.2:**
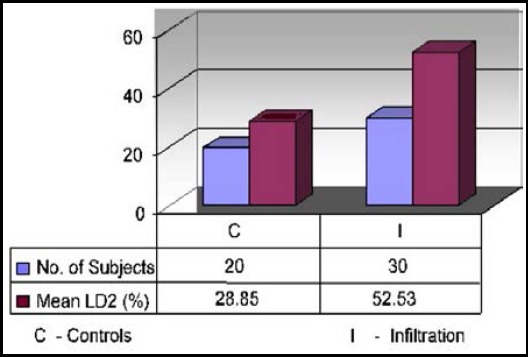
Comparison of Mean Values of LD2 Isoenzyme in Controls and NHL Patients with Marrow Infiltration.

## DISCUSSION

In NHL patients diffuse pattern of bone marrow infiltration was the commonest being observed in 14 (46%) cases followed by interstitial infiltration in 10 (33%) cases. In the focal variety, paratrabecular pattern was present in 4(13%) patients while random type was observed in 2(6%) cases. The diffuse pattern was seen in 9 cases unilaterally and in five cases bilaterally. As regards interstitial pattern it was observed unilaterally in seven cases and bilaterally in three cases while paratrabecular pattern was seen in three cases unilaterally and in one case bilaterally. Random pattern was observed in two cases unilaterally. In the present study markedly raised levels of serological markers i.e.β2m and LD2 were associated with the diffuse pattern, in particular. Similar observations were reported by Mora, Miyashita and Yoo C.^12^-^14^ According to Yoo C and Nakajima Y,^15^,^16^ β2m seems to reflect tumor burden of malignant cells.

As regards patterns of lymphoma infiltration the commonest was diffuse infiltration with marked increase in β2m and LD2 levels followed by interstitial, paratrabecular and random patterns. According to Ding D, Wu L, Yoo C and Rotaru I^17^-^20^ that this observation of β2m and LD2 correlates with the spread of NHL and with possibly poor prognosis in these patients. Diffuse type was the most common pattern of involvement in NHL along with marked increase in β2m and LD2 levels. They observed diffuse pattern in 30% of his subjects which was the most common type. Haddadin^21^ observed diffuse pattern of infiltration in 28% subjects.

Jeong^22^ stated that out of 507 cases of malignant lymphoma, 473 (93.3%) were non-Hodgkin lymphoma (NHL) and regarding bone marrow involvement patterns, diffuse infiltration pattern was the most common (40.0%). Arber, Moid, Gurjal^23^-^25^ also had similar observations in their workup. Overall prognosis was found to be worst with the diffuse type of pattern in their study subjects. So these observations are in agreement with the present study.

### Limitations of the study

The design of our study was cross-sectional i.e., one point in time, so for better observation and proper understanding of the patterns of bone marrow infiltration in NHL patients with β2m and LD2 levels, cohort study should be designed and followed.

## CONCLUSION

By observing the patterns of bone marrow involvement in NHL patients, possible guidelines about prognosis and treatment protocols can be obtained. The serological markers correlate well with the spread of disease and patterns of involvement in NHL patients.

### Authors’ Contribution

**NM:** Conceived, designed and did statistical analysis & editing of manuscript.

**NA:** Reviewed article critically for intellectual content.

**ZI:** Did review and final approval of manuscript.

First author takes the responsibility for intellectual integrity of this study.
